# Effects of high intensity interval training on exercise capacity in people with chronic pulmonary conditions: a narrative review

**DOI:** 10.1186/s13102-020-00167-y

**Published:** 2020-03-30

**Authors:** Abbey Sawyer, Vinicius Cavalheri, Kylie Hill

**Affiliations:** 1 School of Physiotherapy and Exercise Science, Faculty of Health Science, Curtin University, GPO Box U1987, Perth, WA 6845 Australia; 2 Physiotherapy Department, Sir Charles Gairdner Hospital, Perth, WA Australia; 3 Institute for Respiratory Health, Perth, WA Australia; 4 Allied Health, South Metropolitan Health Service, Perth, Australia

**Keywords:** Adults, Chronic pulmonary disease, Exercise, High intensity interval training

## Abstract

**Background:**

Exercise training is important in the management of adults with chronic pulmonary conditions. However, achieving high intensity exercise may be challenging for this clinical population. There has been clinical interest in applying interval-based training as a strategy to optimise the load that can be tolerated during exercise training. Evidence for such an approach is limited in most chronic pulmonary populations.

**Main body:**

In this narrative review, we provide an appraisal of studies investigating whole-body high intensity interval training (HIIT) in adults with chronic obstructive pulmonary disease (COPD). This is the first review to also include studies investigating HIIT in people with conditions other than COPD. Studies undertaken in adults with a chronic pulmonary condition were reviewed when participants were randomised to receive; (i) HIIT or no exercise or, (ii) HIIT or moderate intensity continuous exercise. Data were extracted on peak rate of oxygen uptake (VO_2peak_; ‘cardiorespiratory fitness’) and maximal work rate (W_max_; ‘exercise capacity’).

In people with COPD, two studies demonstrated between-group differences favouring HIIT compared with no exercise. There appears to be no advantage for HIIT compared to continuous exercise on these outcomes. In people with cystic fibrosis (CF), no studies have compared HIIT to no exercise and the two studies that compared HIIT to continuous exercise reported similar benefits. In people prior to resection for non-small cell lung cancer, one study demonstrated a between-group difference in favour of HIIT compared with no exercise on VO_2peak_. In people with asthma, one study demonstrated a between-group difference in favour of HIIT compared with no exercise on VO_2peak_ and one that compared HIIT to continuous exercise reported similar benefits. No studies were identified non-CF bronchiectasis or interstitial lung diseases.

**Conclusions:**

High intensity interval training increases cardiorespiratory fitness and exercise capacity when compared with no exercise and produces a similar magnitude of change as continuous exercise in people with COPD. There is a paucity of studies exploring the effects of HIIT in other chronic pulmonary conditions.

## Background

Exercise training is important in the management of adults with chronic pulmonary conditions. Systematic reviews, undertaken in clinical populations, such as adults with chronic obstructive pulmonary disease (COPD) [1, 2], cystic fibrosis (CF) [3, 4], non-CF bronchiectasis, interstitial lung diseases (ILDs) [5–7], asthma [8], and non-small cell lung cancer (NSCLC) [9–11] have shown that exercise training is effective at increasing components of cardiorespiratory fitness (i.e. the peak rate of oxygen uptake; VO_2peak_) and exercise capacity (i.e. the maximal work rate; W_max_). Additionally, exercise training has been demonstrated to reduce the severity of symptoms experienced during daily life such as dyspnoea and fatigue and improve health-related quality of life [12]. In the general population, exercising at moderate to high intensity is recommended to optimise the magnitude of improvement in cardiorespiratory fitness and exercise capacity [13]. Consistent with these data, studies of people with COPD suggest that, in contrast with low intensity exercise, high intensity exercise training may be advantageous in terms of eliciting a physiological training response [14–17]. These data are in keeping with the overload principle, which states that in order to improve cardiorespiratory fitness and/or exercise capacity, exercise training must be undertaken at an intensity that is greater than the load borne during daily life [18, 19].

Regarding the mechanisms underpinning the improvements in cardiorespiratory fitness and/or exercise capacity, gains are not mediated through improvements in lung function [20–22]. In fact, some of the earliest work done in the area of exercise training for people with COPD [23] was met with scepticism as the gains in exercise capacity were demonstrated without any change in forced expiratory volume in 1 second (FEV_1_). In the mid-1990s, seminal work demonstrated that the changes in exercise capacity were mediated, at least in part, by improved condition of the peripheral muscles, largely vastus lateralis [24]. For example, cycle ergometry training resulted in increased activity of two oxidative enzymes, citrate synthase (CS) and 3-hydroxyacyl-CoA dehydrogenase (HADH) (from 22 ± 4 to 26 ± 4 μmol/min/g muscle for CS, *p* < 0.05, and from 6 ± 3 to 8 ± 3 μmol/min/g for HADH, *p* < 0.01) [24]. In populations characterised by chronic pulmonary conditions, high intensity exercise training may also optimise cardiovascular health [25–27]. Although these data exist predominantly for people with COPD, guidelines regarding the prescription of exercise training for people with other chronic pulmonary conditions often recommend training at moderate intensity or higher [4, 28, 29]. However, achieving high intensity exercise may be challenging for this clinical population. The reasons for this are multi-factorial. First, people with moderate to severe disease are likely to demonstrate ventilatory limitation [14, 30], coupled with worsening pulmonary mechanics during exercise [15], both of which serve to constrain the intensity that can achieved before the onset of intolerable dyspnoea. Second, some people with chronic pulmonary conditions, who do not qualify for long term oxygen therapy, demonstrate a marked reduction in arterial oxygen saturation on exertion. This is due largely to ventilation and perfusion mismatch (V/Q mismatch) and is generally more pronounced in people with severe pulmonary disease [31]. In some clinical populations, such as those with COPD who demonstrate transient exertional desaturation despite being normoxaemic at rest, recent data suggests that, when compared with gains derived from training on room air, the use of supplemental oxygen offers no additional benefit [32]. In other populations, such as those with ILDs, even high dose supplemental oxygen may not be able to prevent marked desaturation [33, 34]. In the presence of transient exertional desaturation, clinicians may choose to reduce the exercise intensity, which compromises the training dose achieved. Third, many people with chronic pulmonary disease, particularly those who are older, have co-morbid conditions which contributes to the difficulty achieving high intensity exercise. These conditions may include osteoarthritis [35], feelings of anxiety and depression [36] or obesity [37]. Given these challenges, there has been clinical interest in applying interval-based training as a strategy to optimise the load that can be tolerated during exercise training [38–40].

### High intensity interval training

Intermittent or interval training approaches are characterised by repeated cycles of ‘work’ interrupted by ‘rest’ [41, 42]. The main difference between these approaches is that, for intermittent training, the patient chooses the work and rest times based on the tolerability of their symptoms, whereas for interval training, the work to rest ratios are prescribed by the therapist. Both approaches are likely to be especially advantageous in people with severe pulmonary disease, who due to intolerable symptoms, may be unable to engage in continuous exercise at an intensity sufficient to induce a training adaptation. In this population, punctuating work periods with rest periods provides intermittent relief from the ventilatory demand associated with exercise, which serves to reduce the work of breathing and dyspnoea. This in turn offers the opportunity to optimise the training intensity that can be borne during the next work period [30]. Given this advantage, the work intervals are often performed at higher intensities than could be tolerated with continuous training. Repeating this work to rest cycle allows the prolonged exposure of the peripheral muscles to high intensity exercise; a stimulus necessary to elicit physiological adaptions [43]. In the literature, interval based training has been described more often than intermittent training because in contrast with an intermittent approach, interval based training is highly standardised, reproducible and the parameters can be manipulated by the therapist. As the work periods are performed at high intensities, this type of training is often described as high intensity interval training (HIIT).

Studies that were reviewed in this paper needed to meet our definition of HIIT; specifically the work intervals used during the training program were conducted at an intensity equivalent to ≥80% of maximum (maximum work rate / peak power / VO_2peak_ or maximum heart rate) determined during a baseline incremental / ramp laboratory-based cycle ergometry test in which work rates progressively increased each minute [13]. As the optimal work to rest ratio for HIIT is unknown, no criteria for inclusion in this review were set for this parameter. Nevertheless, it is worth noting that in order to maximise the stimulus needed to improve the oxidative capacity of the peripheral muscles, a ratio that maximises workload during the work periods and minimises the duration of the rest intervals would seem ideal. In this way, exposure of the peripheral muscles to the milieu of by-products associated with anaerobic metabolism could be maximised; a stimulus necessary to promote mitochondrial biogenesis [43, 44].

One study in people with COPD compared fluctuating work to rest intervals of 4 minutes of work to 4 minutes of rest, using a sinusoidal wave form, with the use of a faster fluctuations, characterised by 1 minute of work to 1 minute of rest. Compared with the slower fluctuations, faster fluctuations between work and rest allowed people with COPD to achieve supramaximal work rates (i.e. 120% of their maximal work rate [W_max_]), with considerably less ventilatory load [43]. This suggests that faster fluctuations between work and rest intervals may offer advantage over slower fluctuations for improvements in muscle adaption, cardiorespiratory fitness and exercise capacity.

Another advantage of HIIT is its efficiency for producing training-related gains. Specifically, in sedentary healthy adults as well as in athletes, this type of exercise produces physiological evidence of a training effect over a period as little as 2 weeks [44]. In healthy young adults (*n* = 8, mean ± SD, aged 22 ± 1 years, peak rate of oxygen uptake [VO_2peak_] 45 ± 3 mL/kg/min), as few as six sessions of cycling-based HIIT over 2 weeks has been shown to produce a significant improvement in endurance capacity (12% change from baseline), and changes in the peripheral skeletal muscles (i.e. vastus lateralis) which were indicative of increased oxidative capacity. These changes were over and above any seen in a control group [45]. However, it is important to note that this study did not show improvements in cardiorespiratory fitness (VO_2peak_), despite improvement in other exercise outcomes.

Importantly, a HIIT protocol may also be more time efficient than a continuous protocol [46]. That is, a randomised controlled trial (RCT) undertaken in sedentary males (*n* = 25, aged 27 ± 8 years, body mass index [BMI] 26 ± 6 kg/m^2^) allocated participants to one of three groups: (i) HIIT (*n* = 9) which comprised three bursts of 20 s cycle sprints interspersed with 2 minute periods of low intensity cycling, completed three times a week for 12 weeks, (ii) continuous cycling (*n* = 10) which comprised 45 min of cycling at ~ 70% maximal heart rate, completed three times a week for 12 weeks or, (iii) a control group (*n* = 6) which did not receive any cycling training. This RCT demonstrated that, compared with the control group, participants in both of the exercise groups increased their W_max_ (mean difference [MD] 62 W, 95% CI 4 to 120 [HIIT] and MD 58 W, 95% CI 7 to 109 [continuous cycling]). However, despite the substantially shorter training time in the HIIT group compared with the continuous exercise training group (30 min a week compared to 135 min a week with workloads titrated according to rate of perceived exertion), the VO_2peak_ on completion of the exercise training period was similar between groups (MD − 2 mL/kg/min, 95% CI − 10 to 6) [46]. The strong physiological rationale for HIIT coupled with data demonstrating both effectiveness and efficiency in sedentary populations makes this approach an attractive option for use in adults with a chronic pulmonary condition. Further, in contrast with other approaches that aim to optimise the training load borne by the peripheral muscles by reducing the ventilatory load associated with exercise (e.g. proportional assist ventilation, heliox), HIIT does not require extra equipment and is an inexpensive option that can be incorporated into daily life.

Recent work has indicated that HIIT is used in clinical practice. That is, our group recently conducted a survey of Australian and New Zealand CF centres to determine, in part, the extent and scope of exercise training in people with CF [47]. The results of this survey found that HIIT is commonly prescribed by therapists despite limited research to support this type of training [47]. In addition, people with interstitial lung diseases (ILDs) are often prescribed interval-based training as a ‘lead in’ phase within studies investigating the effects pulmonary rehabilitation in people with ILDs [48, 49].

The aim of this narrative review was to synthesise the data that have reported the effects of land-based whole-body HIIT on cardiorespiratory fitness (VO_2peak_) and/or exercise capacity (W_max_) in adults living with chronic pulmonary conditions, including people with COPD, CF, non-CF bronchiectasis, asthma, ILDs and NSCLC. This narrative review will provide an update of studies investigating whole-body HIIT in adults with COPD, and will be the first study to review studies in other chronic pulmonary conditions. As HIIT is most often undertaken on a cycle ergometer in studies, outcomes reported on in this review were selected based on the principle of task specificity [50], and comprised VO_2peak_ and W_max_ measured during a cycle-based cardiopulmonary exercise test. Where possible, for each condition, studies that have explored the effectiveness of HIIT compared to usual care (i.e. no exercise) are presented separately to those that have compared the effects of HIIT with continuous exercise training. This is the first review to take this approach.

### Chronic obstructive pulmonary disease

Two studies have compared the use of HIIT, embedded within a 12-week pulmonary rehabilitation program, to no exercise (i.e. the comparison is a control group with no exercise or pulmonary rehabilitation program), on measures of exercise capacity in people with COPD [51, 52]. In one study, the HIIT intervention commenced with ‘work’ intervals at 80% of the W_max_, interspersed with an active recovery (40% W_max_), for 30 and 90 s, respectively, for 20 min, repeated twice per week [51]. When compared with the usual care group (*n* = 15, aged 80 ± 6 years, FEV_1_ 60 ± 15% predicted), those who undertook HIIT (*n* = 14, aged 80 ± 8 years, FEV_1_ 47 ± 18% predicted) demonstrated greater changes in VO_2peak_ (MD 4 mL/kg/min, 95% CI 1 to 7) [51]. In the other study, the HIIT intervention comprised a 45 min session of 30 s intervals of 130 ± 18% W_max_ interspersed with 30 s rest periods, performed thrice weekly [52]. When compared with the usual care group (*n* = 43, aged 67 ± 8 years, FEV_1_ 45 ± 19% predicted), those who undertook HIIT (*n* = 85, aged 65 ± 8 years, FEV_1_ 49 ± 19% predicted) demonstrated greater changes in measures of cardiorespiratory fitness (VO_2peak_; MD 2 mL/kg/min, 95% CI 1 to 4) and exercise capacity (W_max_; MD 16 W, 95% CI 5 to 27) [52]. Nevertheless, as both studies explored the effect of HIIT provided within a pulmonary rehabilitation program, which included resistance exercises and education, the gains in cardiorespiratory fitness and/or exercise capacity cannot be directly attributed solely to the HIIT.

**Fig. 1:**
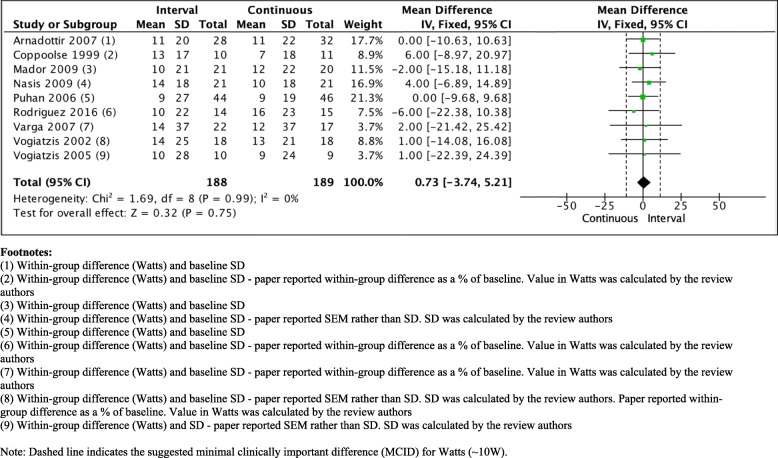
Comparison of effect of HIIT versus continuous exercise training on maximal work rate (measured in Watts)

**Fig. 2:**
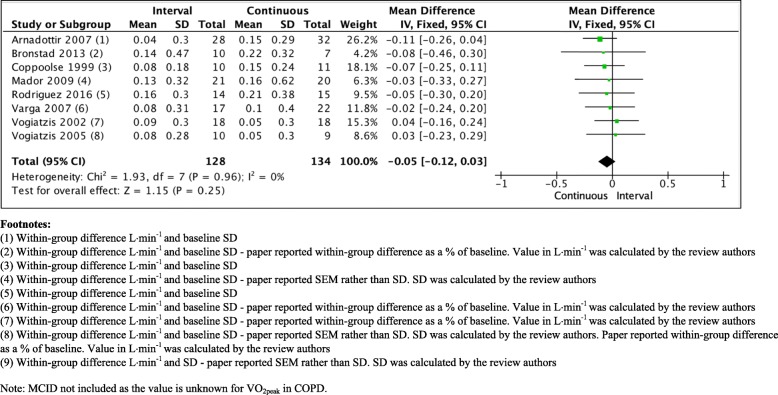
Comparison of effect of HIIT versus continuous exercise training on peak oxygen uptake (measured in L/min)

Several studies have compared the effect of HIIT with continuous training in people with COPD (Table 1). Data from these trials have been meta-analysed in previous reviews [18, 39, 62]. However, we have found a further two trials since a previous comprehensive review and update [39, 62] that compared the effect of HIIT with continuous exercise training on measures of exercise capacity in people with COPD [60, 61]. The findings of the previous comprehensive review demonstrated comparable effects of the two modes of exercise training on measures of cardiorespiratory fitness (VO_2peak_; MD 0.04 L/min, 95% CI 0.13 to 0.05) and exercise capacity (W_max_; MD 1 W, 95% CI − 1 to 3) [39]. Of the 10 studies that have compared HIIT with continuous training, three appeared to be of moderate quality [55, 57, 60], whereas the remaining seven studies were of fair to poor quality [30, 53, 54, 56, 58, 59]. With the addition of the two new studies, the summary effect statistic suggests that, for both outcomes, there was no clear evidence for benefit of one form of exercise over the other (Figs. 1 and 2) [81]. Of note, this result did not differ when studies were grouped according to whether or not the total amount of work between the HIIT group and a continuous group was matched [39], or when a random effects model was used. The authors acknowledge that the updated meta-analysis was not undertaken following a systematic review of the literature, and so may introduce bias to the estimate.

**Table 1:** Description of high intensity interval training compared to continuous exercise training studies in people with chronic obstructive pulmonary disease

	Study	Type of exercise and population	Interval exercise	Continuous exercise	Frequency
Previous review [39]	^a^Arnadottir [53] *n* = 60 2007	Cycling in people with COPD	≥ 80% peak power, followed by 30 to 40% peak power Duration: 39 min (intervals of 3 min: 3 min)	≥ 65% peak power Duration: 39 min	2 x per week for 16 weeks
	Coppoolse [54] *n* = 21 1999	Cycling in people with COPD	90% peak power interspersed with 45% peak power, plus continuous cycling at 60% Duration: 30 min (intervals of 1 min: 2 min)	60% peak power Duration: 30 min	5 x per week for 8 weeks
	Mador [55] *n* = 41 2009	Cycling or treadmill in people with COPD	150% of continuous exercise target interspersed with 75% of target Duration: 20 to 40 min (intervals of 1 min: 2 min)	50% peak power and at 80% of 6MWT average speed Duration: 20 to 40 min	3 x per week for 8 weeks
	Nasis [56] *n* = 42 2009	Cycling in people with COPD	100% peak power and 45% peak power Duration: 30 to 40 min (intervals of 30 s)	60% peak power Duration: 30 to 40 min	3 x per week for 10 weeks
	^a^ Puhan [57] *n* = 98 2006	Cycling in people with COPD	50% peak power of a steep-ramp test, followed by 10% peak power Duration: 25 min (intervals of 20 s: 40 s)	≥ 70% peak power Duration: 25 min	5 x per week for 3 weeks
	Varga [58] *n* = 71 2007	Cycling in people with COPD	50% peak power interspersed with 10% peak power Duration: 45 min (intervals of 20 s: 40 s)	≥ 70% peak power Duration: 45 min	3 x per week for 8 weeks
	Vogiatzis [30] *n* = 36 2002	Cycling in people with COPD	100% peak power and 45% peak power (intervals increased progressively to 140% peak power) Duration: 40 min (intervals of 30 s)	50% peak power, increasing to 70% by the end of the program Duration: 40 min	2 x per week for 12 weeks
	Vogiatzis [59] *n* = 19 2005	Cycling in people with COPD	100% peak power and 45% peak power, (intervals increased progressively to 140% peak power) Duration: 45 min (intervals of 30 s)	60% peak power, increasing to 70% by the end of the program Duration: 45 min	3 x per week for 10 weeks
Additional studies	^a^BrØnstad [60] *n* = 20 2013	Uphill treadmill walking in people with COPD	~ 90% maximal heart rate (‘work’) Duration: 38 min (intervals of 4 min: 4 min)	70% maximum heart rate Duration: 47 min	3 x per week for 10 weeks
	Rodriguez [61] *n* = 29 2016	Cycling in people with COPD	70 to 100% W_max_ (progressively increased over the program), interspersed with 40 to 50% W_max_ Duration: 40 min (intervals of 2 min: 3 min)	60% W_max_ Duration: 40 min	3 x per week for 8 weeks

Abbreviations: *6MWD* six minute walk distance, *6MWT* six minute walk test, *CI* confidence interval, *COPD* chronic obstructive pulmonary disease, *VO*_*2peak*_ peak rate of oxygen uptake, *W*_*max*_ maximum work rate. ^a^ identifies studies whereby the total work undertaken was unmatched between exercise training programs. The studies by Puhan et al. [57] and Varga et al. [58] report prescribing an intensity equivalent to 50% peak power of a steep-ramp test. This protocol is known to produce much higher maximum work rates than the traditional incremental / ramp protocols. In both studies, the authors clarify that work rates equivalent to 50% of the maximum achieved on this test is the equivalent to ~ 90% of maximum work rate achieved during traditional protocols. Rodriguez at al [61] reported that during the first 2 weeks of the program, cycling at high work rate was set to a minimum of 70% W_max_ and was thereafter increased at 5% every week so that by week 3, training was being undertaken at a sufficiently high intensity to be classified as ‘HIIT’

### Cystic fibrosis

Although earlier work suggests that HIIT is feasible in people with CF [38, 63, 64], including those who are characterised by severe expiratory airflow obstruction [38], to date, there are no studies that have compared the effects of HIIT, to no exercise, in this population. Only two studies have compared HIIT with continuous exercise (Table 2). In one study (*n* = 23), where possible, participants completed a continuous training program on a treadmill (aged 26 ± 10 years, FEV_1_ 32 ± 4% predicted) (45 min of exercise at 60 to 70% VO_2peak_, five times per week for 6 weeks) and were only allocated to the HIIT group (aged 26 ± 8 years, FEV_1_ 26 ± 8% predicted) if they were unable to tolerate continuous training. The HIIT program comprised 30 s of walking at the individuals comfortable continuous walking speed (between 3 and 4 km/h) at 50% of the grade achieved during a steep ramp test on a treadmill (modified Balke-protocol) interspersed with 60 s of ‘rest’ (walking at 0% treadmill inclination). This work to rest ratio was repeated 10 times. On completion of the training program, both groups improved their VO_2peak_ (21 ± 4 to 23 ± 7 mL/kg/min in the HIIT group and 21 ± 7 to 25 ± 7 mL/kg/min in the continuous exercise training group; difference in VO_2peak_ between groups was 2 mL/kg/min [95% CI − 5 to 3]). Participants in the HIIT group reported the program to be ‘motivating and less strenuous’ than their previous experience with constant moderate intensity exercise. These results must be interpreted with caution because in this study, group allocation was not decided through a process of randomisation [38]. In addition, while the authors refer to the intervention as HIIT, the equivalent VO_2peak_ and W_max_ achieved during the ‘work; intervals is not described. However, in comparison to previously-mentioned studies in COPD [57, 58], 50% of the intensity achieved on a steep ramp test is equivalent to ~ 90% of maximum work rate achieved during traditional exercise protocols.

**Table 2:** High intensity interval training in adults with cystic fibrosis

Study	Population	Interval exercise	Continuous exercise	Frequency and duration
Gruber [38] *n* = 23 2014	Adults (medically stable inpatients)	1: 2 work recovery ratio (30 s: 60 s, 20 s: 60 s if more deconditioned) on a treadmill. Speed 3 to 4 km/hr. (work) and 50% incline, 0% incline (active recovery) Duration: 16 min (10 interval bouts)	Various sport activities depending on fitness level (i.e. walking, ball games, stretching, balance training and resistance training). HR corresponding 80–90% equivalent to 60 to 75% VO_2peak_ (unmatched workload between groups) Duration: 45 min	5 times x week for 6 weeks
Kaltsakas [65] *n* = 24 2017	Adults	30 s 100% W_max_ interspersed with 40% W_max_ for 30 s Duration: 30 min	70% W_max_ (matched workload between groups) Duration: 30 min	12 weeks (frequency per week not provided)

Abbreviations: *CI* confidence interval, *HR* heart rate, *W*_*max*_ maximal work rate

To our knowledge, only one RCT (available as a conference abstract only) has compared HIIT to moderate intensity continuous exercise in adults with CF (*n* = 24, age and FEV_1_ not reported) [65]. In this study, participants were randomised to 12 weeks of HIIT (*n* = 12), or continuous exercise (*n* = 12). The training programs were matched for total volume of work. The HIIT and continuous exercise group improved their W_max_ by 12% (89 ± 56 W to 108 ± 60 W) and 8% (93 ± 49 W to 109 ± 59 W), respectively. However, although the magnitude of between group change was similar, the 95% confidence interval was wide and offered little precision (MD 1 W, 95% CI − 51 to 49). Similar results were reported for 6 minute walk distance (6MWD), with increases of 45 m (538 ± 70 m to 583 ± 83 m) and 48 m (516 ± 57 m and 564 ± 55 m) in the HIIT and continuous groups, respectively (MD 19 m, 95% CI − 41 to 79) [58]. Despite being matched for the total volume of work undertaken, during the training program, when compared with the continuous group, the HIIT reported lower peak dyspnoea scores (4 ± 1 vs 6 ± 1 [MD 2, 95% CI − 3 to − 1]) and higher nadir oxygen saturation [SpO_2_] (94 ± 1% versus 91 ± 1%, [MD 3, 95% CI 2 to 4]). This suggests that HIIT may be a more tolerable mode of exercise training in adults with CF.

### Non-cystic fibrosis bronchiectasis

There appears to be no published studies that have investigated the effects of HIIT in people with non-CF bronchiectasis.

### Asthma

Although one of the first uncontrolled studies investigating high intensity exercise in people with asthma was undertaken in 1996 [66], to date, only one RCT has evaluated the effect of HIIT compared to usual care (i.e. no exercise) on exercise capacity in this population [67]. In this RCT, untrained people with asthma were allocated to undertake an 8 week intervention period consisting of thrice weekly HIIT (*n* = 20, aged 39 ± 13 years; FEV_1_/FVC 0.91 ± 0.01) on a cycle ergometer, compared with a usual care group (i.e. no formal exercise training) (*n* = 34, aged 38 ± 13 years, FEV_1_/FVC 0.85 ± 0.01). For participants allocated to receive the HIIT, each session comprised a 10 min warm up at a low intensity, followed by consecutive 1 minute exercise bouts for a 5 minute period. The intensity achieved during each minute of exercise was dynamic, with a relative rest and high intensity exercise integrated into each minute bout. That is, the first 30 s was undertaken at < 30% of the maximal heart rate (relative ‘rest’), the second 20 s was undertaken at < 60% of the maximal heart rate, and the final 10 s was undertaken at > 90% of the maximal heart rate (high intensity). The 5 minute sets were repeated twice in the first 2 weeks, and progressively increased up to four sets throughout the intervention period. Each training session concluded with a 10 min cool down. This type of HIIT intervention was reported to be well tolerated and elicited an increase in VO_2peak_ that was over and above any change in the usual care group (between group difference in VO_2peak_ 3 ± 4 mL/kg/min; *p* < 0.0001) [67, 68]. However, the authors speculate that achieving a heart rate of > 90% of the maximal heart rate within a 10 s period may be difficult to replicate in clinical practice. In addition to the effects of HIIT compared to usual care, one RCT (results reported in a conference abstract; *n* = 16 in the HIIT group, no data on age or pulmonary function) demonstrated that HIIT offered comparable benefits to moderate intensity continuous exercise, albeit with lower overall symptoms of dyspnoea [69]. In this study, the HIIT group undertook 30 s work bouts at 80 to 140% of the W_max_. This training regimen was compared with continuous exercise at 70 to 85% of the maximal heart rate. Similar improvements in VO_2peak_ were demonstrated following both modes of exercise (unable to calculate 95% CI from data provided). In addition, dyspnoea was lower during HIIT compared to continuous exercise (*p* < 0.05, unable to calculate 95% CI from data provided).

Nevertheless, one contentious issue regarding HIIT for people with asthma is whether or not it is more or less likely to induce exercise-induced bronchoconstriction. Specifically, some, but not all studies [69] have reported more modest decreases in FEV_1_ in response to HIIT (90% W_max_ for 1 minute followed by 10% of W_max_ for 1 minute, repeated 10 times; FEV_1_–7% ± 8%) when compared with moderate intensity continuous training programs (completed at 65% W_max_; FEV_1_–15% ± 12%) [40, 70]. Similarly, the effects of HIIT compared with continuous training approaches on dyspnoea and rating of perceived exertion experienced during training are also disparate [71, 72]. Studies which included a warm-up period, rather than commencing with work intervals by cycling ‘as fast as possible’ without any resistance on the pedals appeared to induce less bronchoconstriction [70]. When applying HIIT, clinicians should consider offering a warm-up period and monitor FEV_1_ and symptoms for evidence of exercise-induced bronchoconstriction.

### Interstitial lung diseases

Despite the recognised benefit of exercise training for people with ILDs [28, 73–76], there are currently no published studies evaluating the effects of HIIT, compared to usual care or continuous exercise, in people with these conditions. A group of researchers from Melbourne, Australia are currently undertaking an RCT in this field (ACTRN12619000019101). A recent conference abstract reported data collected in a small group of people with ILD (*n* = 6, age and forced vital capacity [FVC] not reported) [77] showing comparable levels of dyspnoea and leg muscle fatigue during HIIT (100% W_max_ for 30 s interspersed with 30 s of unloaded cycling; modified Borg 4 ± 2 and Borg 13 ± 4, respectively) and moderate intensity continuous exercise (60% W_max_; modified Borg 3 ± 1 and Borg 13 ± 5, respectively). Similarly, HIIT was undertaken with comparable exercise heart rates and nadir SpO_2_, despite a higher overall training work load for the HIIT compared to moderate intensity continuous exercise [77].

### Lung cancer

A couple of studies have reported the feasibility and safety of incorporating a component of HIIT in exercise training interventions for people with NSCLC [78, 79]. One RCT compared the effects of short-term HIIT (median [IQR] number of sessions = 8 [7 to 10] in 26 [21 to 33] days) with usual care in people with NSCLC, prior to lung resection surgery [80]. Participants allocated to receive HIIT (*n* = 74, aged 64 ± 13 years, FEV_1_ 86 ± 22% predicted) were asked to attend three supervised exercise sessions per week (variable length of program according to date of surgery). The HIIT comprised a 5 minute warm-up (50% W_max_), following by a 10 min bout of 15 s sprints (80 to 100% W_max_) interspersed with 15 s of low intensity exercise (30% W_max_). This HIIT set was separated by a 4 minute rest, and then repeated (i.e. two 10 min sets were completed per training session). Compared with a control group that received no formal exercise training (*n* = 77, aged 64 ± 10 years, FEV_1_ 88 ± 19% predicted), the HIIT group demonstrated greater improvements in VO_2peak_ on completion of the training program (MD 4 mL/kg/min, 95% CI 2 to 6) [80]. Studies comparing the effects of HIIT with moderate intensity continuous exercise in people with this condition are lacking.

## Conclusions

In people with COPD, when compared with no training, HIIT produces gains in cardiorespiratory fitness and exercise capacity. The magnitude of these gains appears to be similar to those achieved with continuous exercise. In people with CF, non-CF bronchiectasis and ILDs, there are currently no RCTs evaluating the effects of HIIT compared to usual care or to moderate intensity continuous exercise. Nevertheless, in people with CF and ILDs, there are data to show that HIIT is well-tolerated. High intensity interval training in people with asthma is somewhat contentious, owing to the variable effects on bronchoconstriction and symptoms during exercise. Data from one RCT [80] supports the use of pre-operative HIIT (compared to usual care) in people with NSCLC to increase exercise capacity. However, the effects of HIIT compared with moderate intensity continuous exercise in people with NSCLC are unknown.

Some studies (particularly those with the shorter intervals) have shown superior improvement in exercise capacity (workload) with comparable improvements in cardiorespiratory fitness (VO_2peak_) favouring HIIT [53–55, 60, 61]. Additionally, 70% of studies included within this narrative review prescribe interventions whereby the total volume of work was matched between the HIIT and continuous exercise interventions. That is, the overall training load borne was equivalent between the HIIT and continuous exercise, which is likely to explain why similar effects were demonstrated between the two modes of exercise training in most studies. As such, future studies, particularly in COPD, are needed to determine whether HIIT, during which a smaller total training load is prescribed is able to produce comparable or superior benefits in exercise capacity and cardiorespiratory fitness to continuous exercise. This review emphasises the need for large RCTs investigating the effects of HIIT compared to usual care and HIIT compared to moderate intensity continuous exercise in most chronic pulmonary disease populations.

## Data Availability

Upon request from authors.

## Abbreviations

6MWD: Six minute walk distance
6MWT: Six minute walk test
CF: Cystic fibrosis
CI: Confidence interval
COPD: Chronic obstructive pulmonary disease
CS: Citrate synthase
FEV_1_: Forced expiratory volume in 1 s
FVC: Forced vital capacity
HADH: 3-hydroxyacyl-CoA dehydrogenase
HIIT: High intensity interval training
HR: Heart rate
ILD: Interstitial lung disease
IPF: Idiopathic pulmonary fibrosis
IQR: Interquartile range
MCID: Minimal clinically importance difference
MD: Mean difference
NSCLC: Non-small cell lung cancer
RCT: Randomised controlled trial
SD: Standard deviation
SEM: Standard error of the mean
VO_2peak_: Peak rate of oxygen uptake
W_max_: Maximal work rate
